# Novel PVDF-PVP Hollow Fiber Membrane Augmented with TiO_2_ Nanoparticles: Preparation, Characterization and Application for Copper Removal from Leachate

**DOI:** 10.3390/nano11020399

**Published:** 2021-02-04

**Authors:** Mohammed Umar Abba, Hasfalina Che Man, Raba’ah Syahidah Azis, Aida Isma Idris, Muhammad Hazwan Hamzah, Khairul Faezah Yunos, Kamil Kayode Katibi

**Affiliations:** 1 Department of Biological and Agricultural Engineering, Faculty of Engineering, Universiti Putra Malaysia, Serdang 43400, Selangor, Malaysia; gs51611@student.upm.edu.my (M.U.A.); hazwanhamzah@upm.edu.my (M.H.H.); kamil.katibi@kwasu.edu.ng (K.K.K.); 2 Department of Agricultural and Bioenvironmental Engineering, Federal Polytechnic Mubi, Mubi 650221, Nigeria; 3 Smart Farming Technology Research Centre, Level 6, Blok Menara, Faculty of Engineering, Universiti Putra Malaysia, Serdang 43400, Selangor, Malaysia; 4 Department of Physics, Faculty of Science, Universiti Putra Malaysia, Serdang 43400, Selangor, Malaysia; rabaah@upm.edu.my; 5 Materials Synthesis and Characterization Laboratory (MSCL), Institute of Advanced Technology (ITMA), Universiti Putra Malaysia, Serdang 43400, Selangor, Malaysia; 6 Department of Chemical Engineering, Faculty of Engineering, Segi Universiti Malaysia, Petaling Jaya 47810, Selangor, Malaysia; aidaisma@segi.edu.my; 7 Department of Food and Process Engineering, Faculty of Engineering, Universiti Putra Malaysia, UPM, Serdang 43400, Selangor, Malaysia; kfaezah@upm.edu.my; 8 Department of Agricultural and Biological Engineering, Faculty of Engineering & Technology, Kwara State University, Malete, Ilorin 23431, Nigeria

**Keywords:** hydrophilicity, porosity, copper adsorption, nanoparticles, agglomeration

## Abstract

High proportion of copper has become a global challenge owing to its negative impact on the environment and public health complications. The present study focuses on the fabrication of a polyvinylidene fluoride (PVDF)-polyvinyl pyrrolidone (PVP) fiber membrane incorporated with varying loading (0, 0.5, 1.0, 1.5, and 2.0 wt%) of titanium dioxide (TiO_2_) nanoparticles via phase inversion technique to achieve hydrophilicity along with high selectivity for copper removal. The developed fibers were characterized based on scanning electron microscopy (SEM), energy dispersive X-ray spectroscopy (EDX), permeability, porosity, zeta potential, and contact angle. The improved membrane (with 1.0 wt% TiO_2_) concentration recorded the maximum flux (223 L/m^2^·h) and copper rejection (98.18%). Similarly, 1.0 wt% concentration of TiO_2_ nanoparticles made the membrane matrix more hydrophilic with the least contact angle of 50.01°. The maximum copper adsorption capacity of 69.68 mg/g was attained at 1.0 wt% TiO_2_ concentration. The experimental data of adsorption capacity were best fitted to the Freundlich isotherm model with R^2^ value of 0.99573. The hybrid membrane developed in this study has considerably eliminated copper from leachate and the concentration of copper in the permeate was substantially reduced to 0.044 mg/L, which is below standard discharge threshold.

## 1. Introduction

Leachate is a dark aqueous liquid generated from water passing through several layers of waste after undergoing a series of decomposition processes [1,2,3]. Heavy metals have been identified as one of the major constituents of leachate, which are harmful at high concentration with the propensity to accumulate in living organisms [4,5]. Notably, copper is an important trace element among the heavy metals that is essential for the growth of plants, animals, and human being [6]. However, the accumulation of copper above the WHO permissible standard (1.3 mg/L) could trigger environmental and public health hazards such as kidney disorders and severe irritation of the gastrointestinal and nervous system in humans [7]. Excessive amounts of copper have been detected in agricultural soils, animal manures, and swine wastewater [8,9], with the possibility to impede the development of numerous aquatic fauna and flora and present critical difficulties to conventional treatment systems [8,10]. Additionally, high proportion of copper has been reported to possess the ability to ravage the natural environment [11,12,13]. Therefore, the treatment of landfill leachate is essential prior to discharge into natural water ways to mitigate environmental impacts. 

Several techniques have been employed for the removal of copper from wastewater [14,15,16,17]. These techniques include adsorption [18,19], chemical precipitation [20], photocatalysis [21] coagulation-flocculation [22], and electrochemical treatment [23]. Most of these methods are energy consuming, require high operational and maintenance costs, and generate poisonous secondary sludge and liquid waste [24]. Several studies have focused on the adsorptive removal of Cu(II) via membranes [25,26]. The outstanding properties of membrane adsorption, including low energy requirement, small footprint, facile technique, and excellent filtration performance, have made it a suitable technique for the removal of copper from leachate [27,28,29]. However, deposition of foulants on the membrane surface is limiting the application of this technology [30]. Polymers have been the most exploited organic materials in membrane formulation, followed by the inorganic ones (e.g., metals, ceramics, and glass) [31]. Polymeric membranes are likely the most used membranes for water treatment with great design flexibility. Moreover, inorganic membranes, such as ceramic membranes, have high mechanical, thermal, and chemical stability [32]. As a present trend in the field of development of new membrane materials, the merging of both materials to fabricate nanocomposite membranes is also a promising tool for the efficient removal of heavy metals [33]. On this note, adsorptive membranes were fabricated by the use of polymers to enhance adsorption capability of membranes for heavy metals in particular copper removal from water and wastewater [34,35,36]. 

The metal oxide nanoparticles have been frequently used as an additive to increase the membrane performance [37]. Typically, numerous nanoparticles such as ZnO [38,39], TiO_2_ [40,41], Ag_2_O_3_ [42,43], Al_2_O_3_ [44], graphene oxide [45,46], MgO [47], and CuO [48] are frequently employed for the manipulation of a polymeric membranes to augment its hydrophilicity properties. For instance, Song et al. [49] achieved a higher removal of nickel and copper by membrane separation using hydrophilic nanoparticles ion exchange barrier. Bandehali et al. [50] utilizes functionalized glycidyl nanoparticles to remove copper from water and achieved 86% rejection. Hosseini et al. [51] reported higher copper removal from water when activated carbon nanoparticles were used to modify membrane matrix. Kontoudakis et al. [52] removed copper from white wine by membrane filtration and recorded a significant reduction. Amongst the various nanoparticles, TiO_2_ demonstrated a noticeable influence on the performance of the membranes for the removal of metal ions as reported from several studies [41,53,54,55,56]. The introduction of 1.0 wt% TiO_2_ into membrane matrix structure improves the hydrophilicity and makes the surface more negatively charged. On this note, there is going to be electrostatic attraction between the negatively charged membrane surface and the positively charged heavy metals ions, thereby enhancing the adsorption efficacy. The reported impacts were attributed to the great affinity of TiO_2_ toward heavy metals. Despite the potential of the membrane separation technology, the great affinity of TiO_2_ towards heavy metals, and its capacity to increase membrane hydrophilic properties, information on the utilization of TiO_2_ nanoparticles as a potent additive to modify PVDF-PVP membrane for the removal of copper from leachate continues to be very scanty. It is recommended that future research should focus on functionalizing the membrane matrix structure in order to address the problem of agglomeration so as to enhance flux and separation efficacy. In reflection of such interest, the present study fabricates a composite membrane blended with various concentrations of TiO_2_ nanoparticles via phase inversion method. The fabricated fibers were analyzed by means of SEM, EDX, FTIR, zeta potential, porosity, and contact angle. The flux and the copper rejection efficiency from feedwater (leachate) were also examined

## 2. Materials and Method

### 2.1. Experimental Materials

The materials used in the present study include *N*, *N*-dimethylacetamide (Dimethylacetamide (DMAc, Wako Pure Chemical Industries Ltd., Osaka, Japan), which was utilized devoid of additional purification to dissolve the polymer, and PVDF polymer pellets bought from Arkema (Kynar^®^ 760 Inc., Philadelphia, PA, USA). As a co-polymer, PVP was employed to promote the creation of pores and was bought from Sigma-Aldrich (Milwaukee, WI 53209, USA) (MW = 10,000 Da). Sigma-Aldrich (Milwaukee, WI 53209, USA) supplied TiO_2_ (Degussa P25, medium-sized particle size ~21 nm; heat shock pH 7, ≥98% analytic class) was used. The landfill leachate was sourced from a wastewater treatment facility situated in Negeri Sembilan, Selengor, Malaysia, into an airtight container.

#### 2.1.1. Dope Preparation

The procedure employed in the membrane dope preparation has been explained in a previous study [41]. Table 1 present the proportions of solvents, polymers, additives, and nanoparticles used in the dope formulation.

#### 2.1.2. Nano-Composite PVDF-PVP-TiO_2_-Fiber Membrane Spinning

The dope was conveyed into the annular spinneret utilizing the dry-jet wet spinning process [57]. The annular spinneret extrusion needle possessed an internal and external diameter of 0.55 and 1.15 mm. As the inside and outside coagulant, deionized and fresh water was used. The ultimate speed monitor, assembling drum velocity, extrusion rate, air distance, ambient temperature, outer coagulant temperature, and room humidity variables were all kept consistent at 7 rpm, 10 rpm, 5 mL/min, 10 cm, 25 °C, 29.5 ± 1 °C, and 72.7%. In a continuous flow water bath, the spun fibers were then soaked for 24 h to expel all the remaining solvents. Post-treatment was subsequently conducted to cushion constriction by dipping fibers for 12 h into an ethanol solution and then moved for another 5 h into a 10% aqueous glycerol. The fabricated membranes were air-dried at 60 °C for 24 h to ensure complete dehydration. 

### 2.2. Analysis of Fabricated Membrane

#### 2.2.1. Evaluation of Membrane Morphology

At a voltage of 20 Kv, the scanning electron microscopy (SEM) (Model: TM 3000, Hitachi, Tokyo, Japan) was used to capture the digital microstructure of the surface and the cross-sectional area of the whirled membranes. The cross-sectional imaging samples were initially frozen in liquid nitrogen. This is to promote acute fracking and to guarantee a clearer framework. The shattered samples were covered with a slim gold coat and, subsequently, with a carbon tap, placed on the sample holder. The microstructures were subsequently tested with a SEM (S-3400, Hitachi, Tokyo, Japan) at an increased voltage of 20 kV.

#### 2.2.2. Study of Energy Dispersive X-ray Spectroscopy (EDX)

Thermo Scientific was used for EDX assessment of membrane samples with varying TiO_2_ loads; 1 g of fiber samples at various TiO_2_ doses were used for EDX assessment employing SEM (TM 3000, Hitachi, Tokyo, Japan) to analyze the dimensions, dispersion, and elemental constituents of the fibers.

#### 2.2.3. Analysis of Porosity

In determining the porosity of the membrane, the gravimetric approach was employed [40]. Approximately 40 cm of membrane samples were prepared, consisting of five (5) segments each. The open edges of the fibers were closed utilizing epoxy resin and then dipped at ambient temperature (25 ± 1 °C) in distilled water for 5 h. The soaked fibers were gently removed and then, using dry tissue paper, the tracks of water drops on the surface were mopped. The wet membrane (Mw) was used to measure the soaked-mopped fibers. The dipped membranes were then dried-out at 60 °C for 24 h and weighed as dry membrane (*M*_d_). weight. The porosity (*ɛ*) of individual fibers was subsequently calculated utilizing Equation (1) [58,59].

(1) $$\varepsilon \left(\%\right)=\frac{1}{\rho_{w}}\left(\frac{M_{w}-M_{d}}{V}\right)\;\times \;100$$

where *ɛ* is the membrane porosity (%), *ρ_w_* is the density of water, *M*_w_ is the weight of wet membrane, *M*_d_ is the weight of dry membrane, and *V* is the volume of the membrane specimen.

#### 2.2.4. Analysis of Hydrophilicity

The hydrophilicity of the spun fibers was evaluated utilizing a goniometer (OCA 15EC, Data Physics, Succasunna, NJ, USA) relying on the water falling surface contact angle. Initially, a double-sided carbon tape was used, and a dried membrane sample was fastened tightly to the glass plate. Using the goniometer microneedle, approximately 1 µL of distilled water (contact liquid) was released on the membrane surface. The angle of the water droplets was automatically recorded by the instrument. The contact angle was calculated in replicates of 10 for each of the samples and the average mean value was taken into consideration. This method is intended to reduce the degree of data skew.

### 2.3. Membrane Efficiency Assessment

#### 2.3.1. Flux Efficiency

To test filtration or permeability effectiveness, a dead-end filtration system kitted with a membrane module cell was utilized. A peristaltic pump (PLP 6000 produced by Dulabo Laborgeräte, Wertheim, Germany) supplied the membrane with the suction pressure. There are 20 membrane pieces with a uniform length of 35 cm in each of the modules. To ensure steady permeability, the membrane was firstly compressed for 30 min at a pressure of 0.4 MPa, while successive leachate filtration was carried out at a reduced pressure of 0.3 MPa. For a total filtration time of 200 min, the volume of the filtrate accumulated was evaluated at an interval of 50 min. Equation (2) was used to measure the flux of the whirled membranes that separate the pure water (J_w_) and leachate (J_L_).

(2) $$R=\;\left(1-\frac{C_{p}}{C_{f}}\right)\;\times \;100$$

where *R* is the copper removal (%) and *C_p_* and *C_f_* are the copper concentration in the filtrate (mg/L) and feed (mg/L). 

#### 2.3.2. Antifouling and Reutilization Evaluation

The resulting membranes were exposed to 3 filtration cycles with an operational period of 9 h overall. Each filtration cycle was completed under 200 min and was then applied again for an additional filtration cycle after a quick backwash using just 30 min of running tap water. Throughout the experiments, the flux (*J_L_*) was calculated utilizing the corresponding volume of the filtrate obtained at an interval of 50 min. Based on the relative flux recovery (% RFR) and flux recovery ratio (% FRR), as stated in Equations (3) and (4), the antifouling efficiency of the membranes was evaluated [60,61,62,63].

(3) $$\%RFR=\;\left[1-\frac{J_{L}}{J_{w}}\right]\;\times \;100$$

(4) $$\%FRR=\;\left(\frac{J_{w2}}{J_{w}}\right)\;\times \;100$$

where *J_w_* is the water flux, *J_L_* is the leachate flux, and *J_w_*_2_ is the re-evaluated pure water flux after cleaning (all in L/m^2^·h).

### 2.4. Analysis of Zeta Potential

The zeta potential of fabricated membranes was assess utilizing an electrokinetic analysis device from Anton Paar SurPASS (Berlin, Germany) using a flexible gap cell. The measurements were conducted utilizing an electrolyte solution refined at 0.1 M KCl with nitrogen. To change the pH level, 0.1 M NaOH and 0.05 M HCl were employed during the measurements.

### 2.5. Analytical Technique

Before and after treatment, the bicinchoninate method was used to evaluate the copper concentration present in the leachate. A 10 mL of leachate was filled into a sample cell. Cu Ver 1 copper reagent powder pillow was augmented to the sample cell. Within 30 min after the timer sounds the fabricated sample was inserted into the cell holding device of the ultraviolet–vis spectrophotometer (DR/4000u HACH, Loveland, CO, USA) at a wavelength of 560 nm to assess the concentration of copper in the leachate sample. Distilled water used in the experiments was sourced from the Milli-Q water refining device (18 MQ cm).

## 3. Results and Discussion

### 3.1. Impact of TiO_2_ on Membrane Physical Properties

#### 3.1.1. EDX Elemental Evaluation

**Figure 1:**
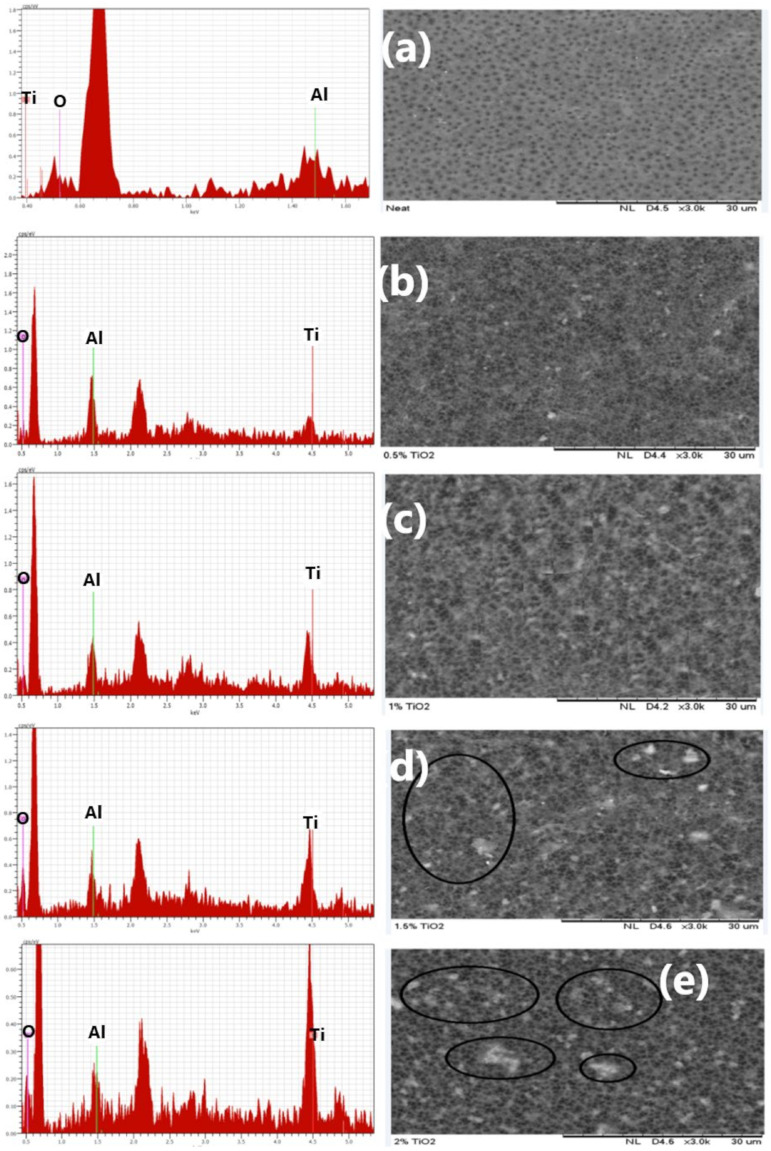
Matrix structure and elemental constituents of (**a**) neat TiO_2_, (**b**) 0.5 wt% TiO_2_, (**c**) 1.0 wt% TiO_2_, (**d**) 1.5 wt% TiO_2,_ and (**e**) 2.0 wt% TiO_2_.

Figure 1 exhibits the matrix structure and elemental constituents of the fabricated fibers. The neat membrane (Figure 1a), as shown in the elemental composition, has only oxygen and carbon on its structure, while membranes accreted with a TiO_2_ dose of 0.5 wt% (Figure 1b) showed C, O, and Ti elemental composition. It was observed that with a rise in TiO_2_ dosage in the dope, the Ti fraction in the elemental composition increased. Nevertheless, the O compositions decreased at greater TiO_2_ dosage (2.0 wt%) as depicted in Figure 1e. As shown in Figure 1b,c, a free agglomeration is presented at 0.5 and 1.0 wt% TiO_2_ concentration. Fibers with 1.5 and 2.0 wt% TiO_2_ dosages (Figure 1d,e), on the other hand, exhibit heterogeneous dispersion and particle fragments, leading to agglomeration and viscosity upsurge in the dope [64].

#### 3.1.2. Morphological Structures

**Figure 2:**
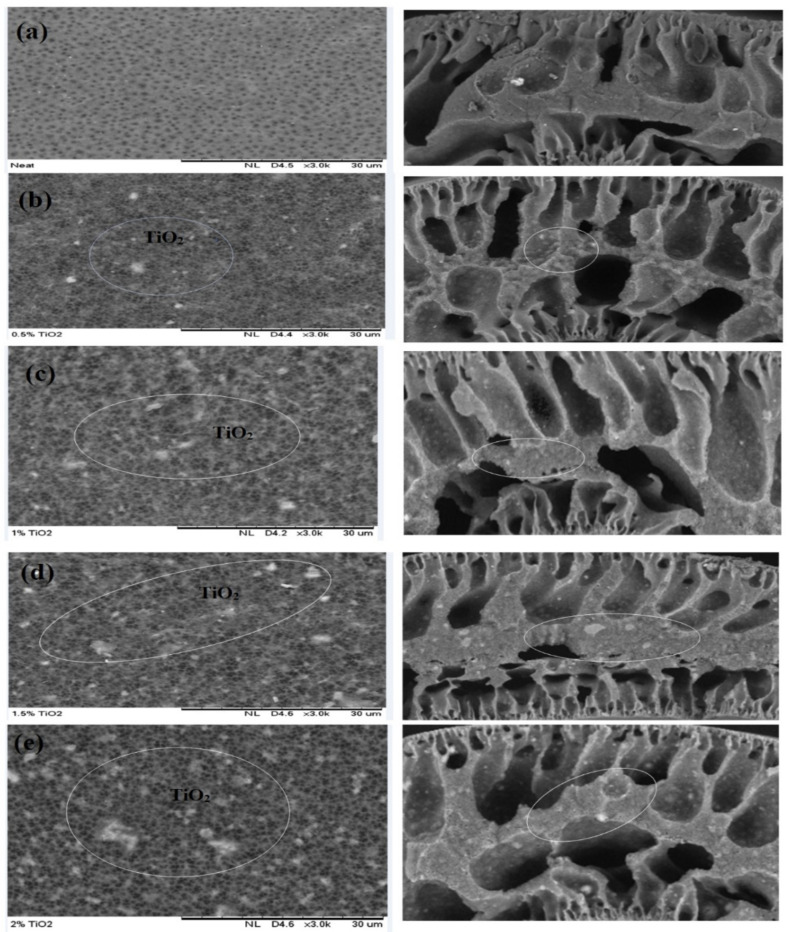
Scanning electron microscopy (SEM) images (cross and outer surface) of membranes fabricated with varying concentrations of TiO_2_: (**a**) neat TiO_2_, (**b**) 0.5 wt% TiO_2_, (**c**) 1.0 wt% TiO_2_, (**d**) 1.5 wt% TiO_2_, and (**e**) 2.0 wt% TiO_2_.

The scanning electron microscopy of the cross-section of different membranes fabricated with various TiO_2_ doses is demonstrated in Figure 2. The incorporation of TiO_2_ nanofillers into a membrane dope produces larger pores on the membrane matrix [65]. The finger-like pores of the improved membranes become bigger with increasing TiO_2_ loading (0.5–2.0 wt%) as shown in Figure 2b,e [47]. This is attributed to the nucleation effects together with crosslinks produced amidst the TiO_2_ and the polymeric materials [47]. However, from 0.5 to 1.0 wt%, uniform distribution of TiO_2_ on the membrane matrix was observed as depicted in Figure 2a,c. Conversely, these nanoparticles appeared to agglomerate at a higher TiO_2_ dosages (1.5 and 2 wt%) as shown in Figure 2d,e, and produced larger nanofillers, resulting in pore stoppage and decrease in water flux [66].

#### 3.1.3. Evaluation of Hydrophilicity

**Figure 3:**
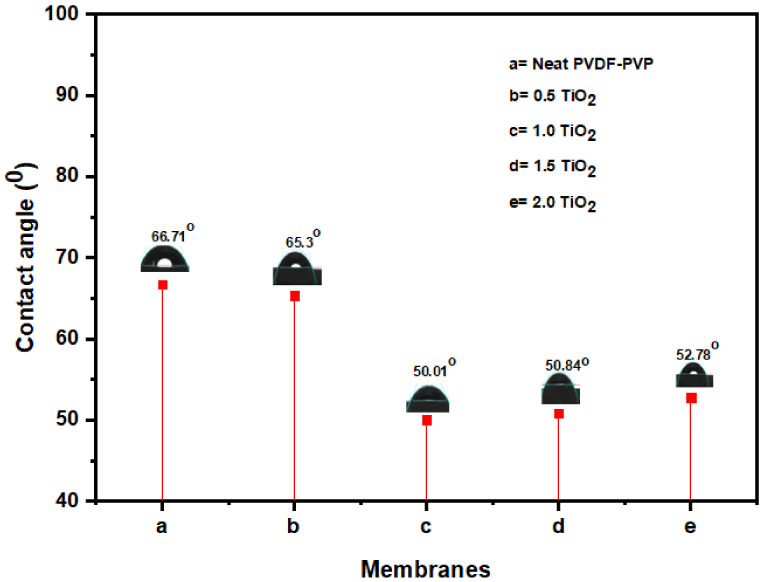
Membrane contact angle at various TiO_2_ loading: (**a**) neat TiO_2_, (**b**) 0.5 wt% TiO_2_, (**c**) 1.0 wt% TiO_2_, (**d**) 1.5 wt% TiO_2_, and (**e**) 2.0 wt% TiO_2_.

Membrane surface hydrophilicity can be evaluated by determining the contact angle [67]. Figure 3 shows the contact angle of a neat and improved fibers. A strong distinction of both hydrophilicity and flux was rendered by the incorporation of TiO_2_ nanofillers into the polymer dope solution [67]. At 1.0 wt% TiO_2_ concentration, the modified membrane demonstrated the most hydrophilic with least contact angle of 50.01°, while the un-modified membrane recorded 66.7°. Further rise in TiO_2_ dosage (1.5 and 2.0 wt%) results in the accumulation of nanoparticles inside the membrane matrix. This can be due to a heterogeneous dispersion of nanoparticles, a reduction in the surface potential, and a clogging of membrane pores [68,69]. The findings of the present and previous investigations revealed that the existence of TiO_2_ nanoparticles in the matrix structure of the improved fibers enhances its hydrophilicity [41]. In addition, scholars have also revealed that most polymer membranes with strong hydrophilic characteristics are more likely to survive fouling [65,70].

#### 3.1.4. Zeta Potential

**Figure 4:**
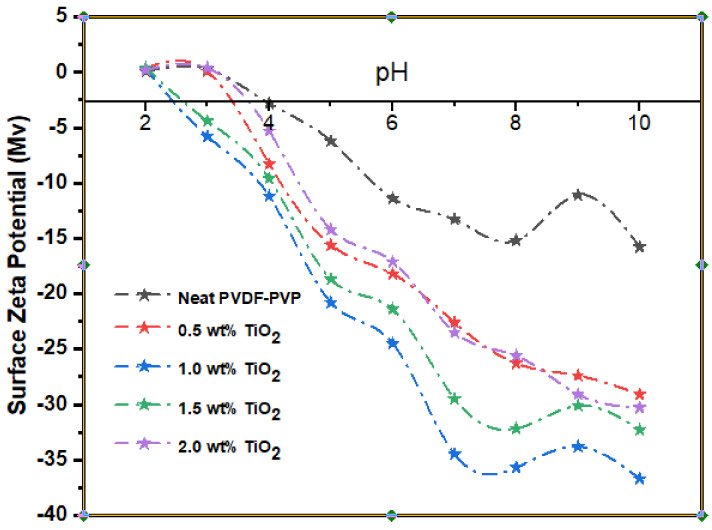
Membrane surface zeta potential at varying TiO_2_ loading (0, 0.5 wt%, 1.0 wt%, 1.5 wt%, and 2.0 wt%).

The surface charge of the neat and modified membranes was measured by a zeta potential analyzer. The surface zeta potential of the resultant membrane at various ranges of 2–10 pH is shown in Figure 4. Alongside increasing TiO_2_ loading, the membrane matrix was more negatively charged. The TiO_2_ exposed to the membrane surface has been hydrolyzed in the presence of water to form a functional hydroxide group, as depicted in Figure 4 TiO_2_ protonation has led to deprotonation of the membrane surface [71]. Visibly, 1.0 wt% TiO_2_ membrane had maximum negative zeta potential charge at pH 10 with −37 Mv. The hydrophilicity and zeta potential are critical variables in membrane analysis. The surface zeta potential of a membrane offers information about the membrane matrix surface charges which relies on the feed stream quality.

#### 3.1.5. Membrane Porosity

The result of membrane porosity has been described in our previous studies [41]. Based on this result, the porosity of the membrane is significantly influenced by TiO_2_ loading rate in the dope solution. Additionally, the modified fibers with 1.0 wt% TiO_2_ dosage exhibit superior porosity. Though, additional TiO_2_ dosage (1.5–2.0 wt%) led to a decrease in porosity owing to rise in dope viscosity with lower pore volume [72,73]. The membrane with 2.0 wt% TiO_2_ dosage had the least porosity among modified membranes. This may be attributed to the conglomeration and higher viscosity impact on the dope [74]. 

#### 3.1.6. Permeability Flux

The effect of TiO_2_ loading (0–2.0 wt%) on the membrane flux has been described in our previous studies [41]. It was noted that the flux for water and leachate filtration decreased to 207 and 156 L/m^2^·h at a higher load of TiO_2_ nanoparticles (1.5 wt%). This is attributed to the nanoparticles’ agglomerating effect on the membrane matrix [47], and heterogeneous dispersion of nanoparticles and obstruction of micropores on the surface of the membrane [68,69]. Moreover, the presence of agglomerated particles in the matrix structure could distort the additive’s surface area interface (TiO_2_) and initiate more roughness on the exterior surface of the [66]. Similarly, the membrane flux could be undermined due to the clogging of pores that impede water passage [66]. Generally, relative to pure water flux, the amount of flux in leachate was considerably lower due to the influence of more contaminants, particles, and colloidal objects in the leachate [38]. 

#### 3.1.7. Copper Removal

**Figure 5:**
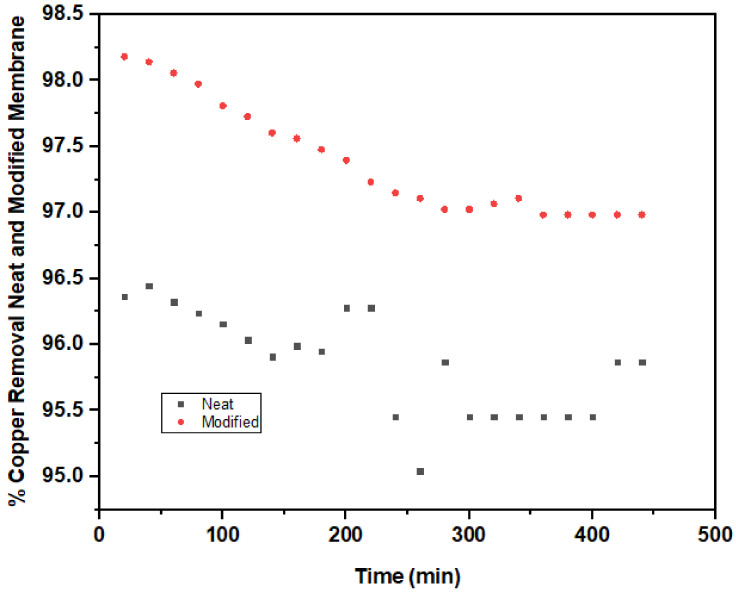
Copper removal by neat (PVDF-PVP) and modified (1.0 wt% TiO_2_) membrane.

The percentage of copper removal using neat and modified membranes under steady scouring aeration of 5 L/min is presented in Figure 5. The neat membrane had 96.36% copper removal in the first 20 min of filtration. At the initial filtration time, the removal performance fluctuated between 80–300 min. Thereafter, the percentage of copper removal remained stable at 340–400 min of filtration with 95.45% copper removal efficiency. 

A significant improvement was noticed in the percentage of copper removal with 98.18% with a corresponding final concentration of 0.044 mg/L, which is far below 1.3 mg/L of WHO standard discharge limit for copper. Additionally, a steady removal performance was maintained at 360–440 min filtration duration with 96.98% removal rate. The percentage of copper removal recorded in the present study is in good agreement with the result obtained from [75] when a combined membrane filtration and electrodialysis treatment was used to remove copper from wafer polishing wastewater. Ghaemi et al. [74] introduced PPy@Al_2_O_3_ nanoparticles into polyethersulfone (PES) flat sheet membrane and significantly elevated copper removal during the membrane filtration.

#### 3.1.8. Copper Removal Mechanism

**Figure 6:**
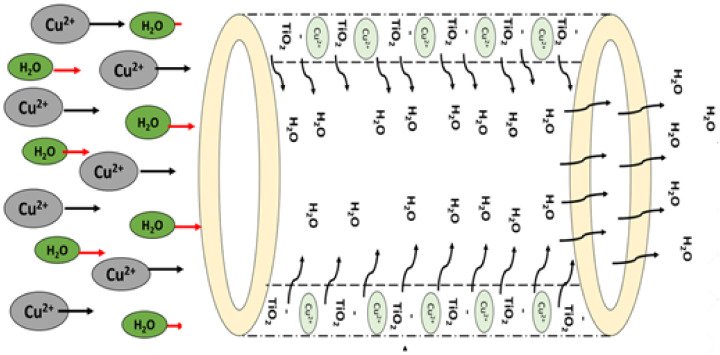
Copper removal mechanism on membrane matrix structure.

The introduction of nanofillers onto the membrane matrix tailored the surface slightly more negatively [76,77]. The electrostatic attraction between the positively charged copper ions and the negatively charged membrane surface enhances copper adsorption. The positively charged copper ions are attracted to the negatively charged fiber matrix as depicted in Figure 6. The higher dispersion of nanoparticles on the membrane matrix led to an increase in the available sorption sites on the membrane surface [78]. The membranes could operate as an adsorptive barrier owing to the chemical structure of nanofillers, the augmentation of available active sites, and higher surface area determine the cation removal performance. The heavy metal ions in the feed solution can be captured by the adsorbent though the physical or chemical adsorption [79]. The treated permeate is collected and then subjected to analysis. 

#### 3.1.9. Adsorption of Copper by Modified Membrane

**Figure 7:**
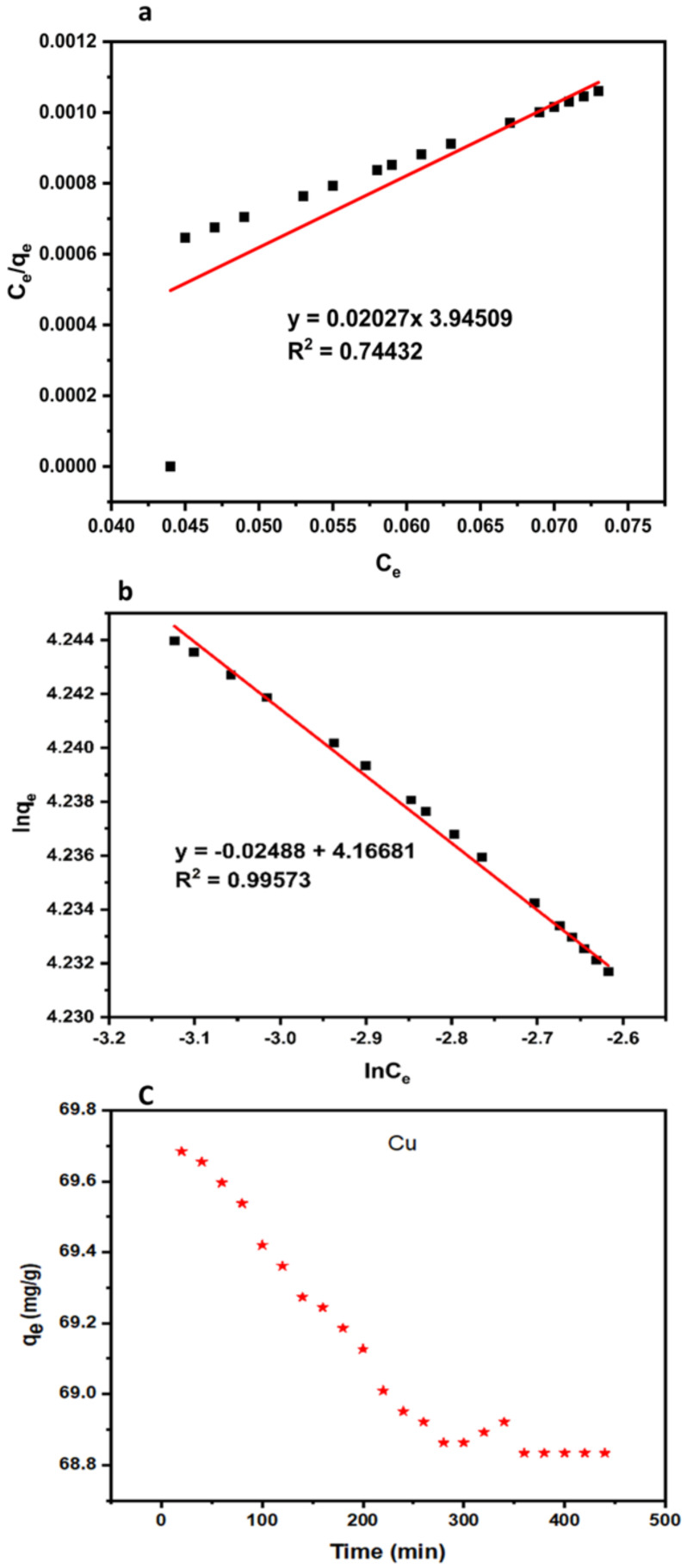
Langmuir (**a**), Freundlich isotherm, (**b**) and copper adsorption capacity (**c**) into hollow fiber membrane at pH 10, time 440 min, and initial copper concentration 2.42 mg/L.

Adsorption is strongly linked to membrane pore radius, which permits most heavy metals with molecular weight less than the molecular cut off to access and diffuse into the membrane internal adsorption sites [80]. The occurrence of this mechanism is therefore mainly based on the amount of available adsorption sites at the surface of the membrane, together with the hydrophobicity of the compound. As earlier elucidated, the improved fiber with 1.0 wt% dosage is the most hydrophilic among the improved membranes. Hence, the adsorption capacity of copper into the modified membrane (1.0 wt% TiO_2_) was studied as demonstrated in Figure 7c. The first 20 min filtration period recorded the maximum adsorption capacity (Q_max_) of 69.68 mg/g. The result obtained is attributed to the availability of active pore sites, which enhances the adsorption process. The higher adsorption capacity recorded in this study was consistent with the findings from [26]. The adsorption capacity had a slight decline and remained stable after 340 min filtration period. The slight reduction in adsorption capacity was due to membrane fouling. The experimental data of adsorption capacity were best fitted to the Freundlich isotherm model with an R^2^ value of 0.99573 as depicted in Figure 7b.

#### 3.1.10. Evaluation of Membrane Fouling

**Figure 8:**
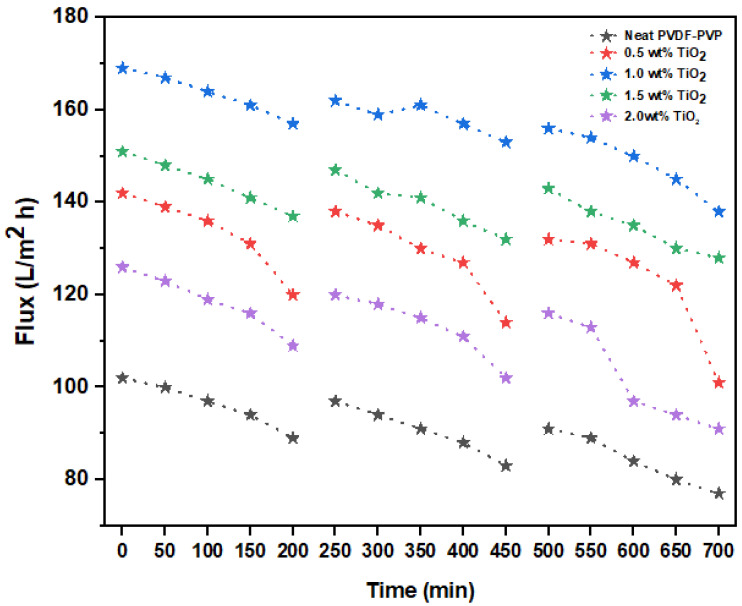
Antifouling characteristics of neat and modified membranes (0.5, 1.0, 1.5, and 2.0 wt% TiO_2_).

The effects of flux in three filtration cycles with regard to time are shown in Figure 8. Noticeably, there was a decrease in flux for all the fabricated membranes, including membranes modified with varied TiO_2_ dosages (0–2.0 wt%) over time as a result of the accumulation of foulants on the surface of the membranes. The unmodified fibers had a flux of 89 L/m^2^·h, while the transformed membrane with 1.0 wt% TiO_2_ recorded a flux of 157 L/m^2^·h after 200 min of operation. A decline in permeability flux of 77 and 138 L/m^2^·h was noticed for the neat and modified membranes, respectively. Hydrophobic membranes are much more vulnerable to fouling due to the effective adhesive attraction between the collaborating interfaces [69]. Foulant deposition can be resolved by incorporating hydrophilic nanofillers into the membrane matrix. The membranes were substantially washed for 30 min after each filtration cycle under flowing tap water.

#### 3.1.11. Performance Evaluation with Literature

Information on comparison of the current study with previous studies on adsorptive removal of heavy metals by membrane is provided in this section. The result of the present study revealed that dispersion of 1.0 wt% TiO_2_ concentration into PVDF-PVP dope improves the membrane performance in terms of contact angle (50.01°), flux (223 L/m^2^·h), and copper adsorption capacity (69.68 mg/g). Mundal and Kumar [81] fabricated polysulfone-based hybrid ultrafiltration membranes for the adsorptive removal of Pb^2+^ ions from polluted aqueous solutions; 279.63 mg/g was obtained as the highest adsorptive capacity for the feed concentration of 200 mg/L, and the permeate flux of 1.65 mL min^−1^. However, despite higher adsorption capacity, the permeate flux is relatively low. Additionally, Abdullah et al. [82] utilized polysulfone/hydrous ferric oxide ultrafiltration mixed matrix membrane for the adsorptive removal of lead (II) from aqueous solution. The result of the study showed that the highest adsorption capacity of Pb(II) was 13.2 mg/g. Similarly, Adam et al. [83] employed novel natural zeolite based hollow fiber ceramic membrane for the adsorptive removal of chromium (VI) in aqueous solution. The performance of the resultant membrane in adsorption-filtration was 44% of Cr (VI) removal at the Cr (VI) concentration of 40 mg/L and pH 4. However, the copper removal efficiency was very low.

A comparative study on the adsorptive removal of copper by membrane in the present study and other literatures is presented in Table 2. The high adsorption capacity recorded in the present study could be attributed to the small pore size and high surface area of the TiO_2_ nanoparticles. The present study fills a knowledge gap, considering the fact that information on the utilization of TiO_2_ nanoparticles as a potent additive to modify PVDF-PVP membrane for the removal of copper from leachate continues to be very scanty.

## 4. Conclusions

This study demonstrated the preparation and characterization of PVDF-PVP hollow fiber membrane impregnated with TiO_2_ nanoparticles and their potential ability to remove copper from leachate. The nanoparticles improve the membrane negative surface potential and enhance their permeability due to increased membrane porosity. The fabricated membrane blended with TiO_2_ nanoparticles at varied concentrations (0–2.0 wt%). Notably, fibers improved with 1.0 wt% dosage exhibited higher flux of 223 L/m^2^h and 172 L/m^2^h for pure water and leachate. The high porosity of 85.50% recorded at 1.0 wt% loading of TiO_2_ nanoparticles into the membrane matrix resulted in improvement in surface hydrophilicity relative to the pristine membrane. Furthermore, the modified membrane (1.0 wt%) is more hydrophilic with the least contact angle of 50.01° and superior copper rejection (98.18%) together with higher copper adsorption capacity of 69.69 mg/g. The experimental data of adsorption capacity were best fitted to the Freundlich isotherm model with an R^2^ value of 0.99573. The present study concluded that the nanoparticle additives improve the membrane porosity, flux, copper adsorption and rejection, hydrophilicity, and antifouling properties. Hence, the developed hybrid PVDF-PVP membrane modified with 1.0 wt% TiO_2_ can be successfully used for the treatment of industrial effluent-containing copper ions. 

## Figures and Tables

**Table 1:** Dope chemical constituents.

Membrane Constituents	Solvent (DMA_C_) (wt%)	Polymer (PVDF) (wt%)	Additive PVP (wt%)	TiO_2_ (wt%)
pure	78.0	19.0	3.0	0.0
(0.5)	77.5	19.0	3.0	0.5
(1.0)	77.0	19.0	3.0	1.0
(1.5)	76.5	19.0	3.0	1.5
(2.0)	76.0	19.0	3.0	2.0

**Table 2:** Comparison of adsorptive removal of heavy metals by membrane.

Membrane	Removal Mechanism	Pollutant	q_e_ (mg/g)	Re (%)	Remark	Reference
Hollow fiber	Adsorption	Cu^2+^	92.38	NA	The Langmuir isotherm model best fitted the adsorption isotherms	[84]
Polysulfone Ultrafiltration	Adsorption	Cu^2+^	279.63	ND	Impressive adsorption capacity	[81]
Ultrafiltration membrane	Adsorption	Cu^2+^	2.82	97	The membrane has removed Cu (II) from water at low pressure	[85]
Adsorptive membranes	Adsorption	Cu^2+^	20.1		The results suggested that the membrane can remove copper	[86]
PES modified membrane	Adsorption	Cu^2+^	NA	92	Higher copper removal achieved	[87]
PVDF/ZnO hybrid membranes	Adsorption	Cu (II) ion	11	ND	Better adsorption and desorption properties for copper ions	[88]
PVDF-PVP-TiO_2_	Adsorption	Cu^2+^	69.68	98.18	WHO standard achieved	Present study

NA = Not available.

## Data Availability

Not applicable.
